# Is video gaming, or video game addiction, associated with depression, academic achievement, heavy episodic drinking, or conduct problems?

**DOI:** 10.1556/JBA.3.2014.002

**Published:** 2014-02-03

**Authors:** Geir Scott Brunborg, Rune Aune Mentzoni, Lars Roar Frøyland

**Affiliations:** ^1^Norwegian Institute for Alcohol and Drug Research, Oslo, Norway; ^2^Department of Psychosocial Science, University of Bergen, Bergen, Norway; ^3^Norwegian Social Research, Oslo, Norway

**Keywords:** video game, addiction, consequences, outcomes, longitudinal, adolescent

## Abstract

*Background and aims:* While the relationships between video game use and negative consequences are debated, the relationships between video game addiction and negative consequences are fairly well established. However, previous studies suffer from methodological weaknesses that may have caused biased results. There is need for further investigation that benefits from the use of methods that avoid omitted variable bias. *Methods:* Two wave panel data was used from two surveys of 1,928 Norwegian adolescents aged 13 to 17 years. The surveys included measures of video game use, video game addiction, depression, heavy episodic drinking, academic achievement, and conduct problems. The data was analyzed using first-differencing, a regression method that is unbiased by time invariant individual factors. *Results:* Video game addiction was related to depression, lower academic achievement, and conduct problems, but time spent on video games was not related to any of the studied negative outcomes. *Discussion:* The findings were in line with a growing number of studies that have failed to find relationships between time spent on video games and negative outcomes. The current study is also consistent with previous studies in that video game addiction was related to other negative outcomes, but it made the added contribution that the relationships are unbiased by time invariant individual effects. However, future research should aim at establishing the temporal order of the supposed causal effects. *Conclusions:* Spending time playing video games does not involve negative consequences, but adolescents who experience problems related to video games are likely to also experience problems in other facets of life.

## Introduction

Several studies have shown that use of video games is associated with a host of different problems (Griffiths, Kuss & King, 2012). Previous studies have for instance shown that the amount of time spent on video games is associated with higher levels of depression (Lemona et al., 2011), lower academic achievement (Anand, 2007; Gentile, Lynch, Linder & Walsh, 2004), more alcohol consumption (Ream, Elliott & Dunlap, 2011), and conduct problems (Holtz & Appel, 2011). This suggests that amount of gaming may be predictive of negative outcomes. However, studies have also found that time spent on video games is *not* related to negative outcomes (e.g. Desai, Krishnan-Sarin, Cavallo & Potenza, 2010; Ferguson, 2011; Ferguson, San Miguel, Garza & Jerabeck, 2012; von Salisch, Vogelgesang, Kristen & Oppl, 2011). This suggests that amount of gaming in itself is not necessarily associated with detrimental effects. There is greater agreement that experiencing problems related to gaming is related to other negative consequences. For instance, research has shown that video game *addiction* is associated with depression (Gentile et al., 2011; Mentzoni et al., 2011), poor academic achievement (Skoric, Teo & Neo, 2009), alcohol use problems (Ream et al., 2011), and conduct problems (Rehbein, Kleinmann, Mediasci & Möβle, 2010).

Despite a great number of studies focusing on video game addiction, there is still lack of consensus concerning which terms to use, how the phenomenon should be defined, and what methods should be used to measure it. Based on a review of the literature, King, Haagsma, Delfabbro, Gradisar and Griffiths (2013) proposed that the core features of video game addiction are withdrawal symptoms experienced when not able to play video games, loss of control over how much time is spent on video games, and conflict in terms of personal relationships and school/work commitments arising from video gaming. Video game addiction is not recognized as a formal psychiatric diagnosis, but it is listed as a condition for further study in the recently published fifth version of the Diagnostic and Statistical Manual of Mental Disorders (American Psychiatric Association, 2013). In the ongoing debate about how video game addiction should be conceptualized, it has been suggested that high engagement should be distinguished from addiction (e.g. Charlton & Danforth, 2007). The possible negative impact of time spent on games may be dependent on individual and contextual factors. Therefore, researchers have started to differentiate between enthusiasm for video games and problems associated with gaming (Brunborg et al., 2013; Charlton & Danforth, 2007; Ferguson, Coulson & Barnett, 2011; Rehbein et al., 2010; Skoric et al., 2009). The emerging evidence suggests that video game addiction is associated with negative consequences, but that high engagement with games is not (Brunborg et al., 2013; Ferguson et al., 2011; Skoric et al., 2009).

This field of research still has some way to go before claims can be made about the direction of causality between use of video games and negative outcomes. One complicating factor is that the reported findings could in many cases be explained by unmeasured third variables. For instance, the reported relationship between video gaming and conduct problems could be explained by high sensation seeking. Research has shown that sensation seeking may be related to both video gaming and rule-breaking behavior (Jensen, Weaver, Ivic & Imboden, 2011). Also, the relationship between video gaming and depression could be explained by trait anxiety (Mentzoni et al., 2011). Researchers sometimes attempt to control for third variables (e.g. gender, age, and socio-economic status, intelligence, personality) by including such variables in regression models. However, as there will always be omitted variables, this approach may be insufficient and cause biased estimates (Verbeek, 2012). One method for dealing with such omitted variable bias is first differencing (FD). FD requires data with more than one observation of the same individuals (panel data), and provides a safeguard against bias resulting from omitted time-invariant individual variables (Allison, 1990; Nordström & Pape, 2010; Wooldridge, 2001). The FD method is described in the statistics section below.

In the current study, we investigated the relationships between amount of time spent on video games and several possible negative outcomes (depression, poor academic achievement, alcohol intoxication and conduct problems), as well as the relationships between video game addiction and the same negative outcomes. The current study is the first to use FD to investigate the relationship between video game use and associated problems. Therefore, our study is the first to control for all possible time invariant individual variables when investigating the negative outcomes of video gaming.

## Methods

### Data

This study used data from the surveys “Young in Norway 2010” and “Young in Norway 2012”, where the goal was to collect the same information from the same individuals at two time points separated by two years. In 2010 (t1), the survey was administered at a total of 89 schools in Norway. The sample of schools were drawn in order to gain a representative sample of Norwegian adolescents, and included elementary schools (final year only when children are 12 years old), junior high schools (age range 12 to 16 years), and senior high schools (age range 16 to 19 years). A total of 11,487 students were invited to participate in the survey. Of these, 8,356 students participated, which equals a response rate of 72.7%. The questionnaires were completed in one school hour, and students not present at the time of the data collection were given the chance to complete the survey at a later occasion.

In 2012 (t2), 4,561 of the adolescents who took part in the 2010 survey were invited to take part in a follow-up survey. There were two reasons why not all respondents from the 2010 survey were invited, 1) they had not consented to be invited for the follow-up (*n* = 2,021), and 2) contact information was missing (*n* = 1,774). Among those invited, 2,450 participated, which equals a response rate of 53.7%, however, the proportion who participated in 2012 of those who participated in 2010 was 29.3%.

The questionnaire that was administered at the elementary schools in 2010 was an abbreviated version of the questionnaire administered at junior high and senior high schools. This version did not include the variables of interest to the current study. Consequently, the elementary school students were not included in the analytical sample.

The analytical sample used in the current study consisted of 1,928 adolescents (55.5% female), with an age range of 13 to 17 years in 2010.

## Measures

*Video game use.* Responses to two questions were used to estimate time spent gaming in the last four weeks. One question asked about the duration of a typical gaming session (scored 0 = “usually don’t”, 0.5 = “less than 1 hour”, 1.5 = “1–2 hours”, 2.5 = “2–3 hours”, 3.5 = “3–4 hours”, 5 = “4–6 hours”, and 7 = “more than 6 hours”. The other question asked about the frequency of gaming (scored 0 = “never, or almost never”, 2 = “1–3 days a month”, 4 = “one day a week”, 14 = “several days a week”, and 30 = “daily or almost daily”). Time spent gaming was the product of the quantity and frequency questions. Scores ranged from 0 to 210.

*Video game addiction.* The seven-item version of the “Game Addiction Scale for Adolescents” (Lemmens, Valkenburg & Peter, 2009) was used to measure video game addiction. Each of the seven items measures one of the DSM criteria for addiction: Salience, tolerance, mood modification, withdrawal, relapse, conflict, and problems. A five-point scale (1 = “never”, 2 = “almost never”, 3 = “some-times”, 4 = “often”, 5 = “very often”) was used for respondents to indicate how often each event had occurred in the past six months. The averages of the seven scores were used in the analysis (range 1–5). Cronbach’s alpha for the scale in the current study was .86 at t1, and .90 at t2.

*Depression.* Depression was measured using six items derived from the Hopkins Symptom Checklist (Derogatis, Lipman, Rickels, Uhlenhut & Covi, 1974). Respondents were asked to indicate the degree to which they had experienced the following six complaints during the previous week: “Felt too tired to do things”, “had trouble sleeping”, “felt unhappy, sad, or depressed”, “felt hopeless about the future”, “felt tense or keyed up”, and “worried too much about things”. Responses were made on a four point scale (1 = “not distressed at all”, 2 = “a little bit distressed”, 3 = “quite a bit distressed”, and 4 = “extremely distressed”). Average scores ranging from 1 to 4 were used in the analysis. Cronbach’s alpha for the scale was .85 at t1, and .87 at t2.

*Academic achievement.* Respondents indicated the grades they had received for three subjects last time they received a school report card. The subjects were Written Norwegian, Mathematics and English. Grades in Norway range from a maximum of 6 to a minimum of 1, where 1 indicates a fail. The average of the three grades was used as an indicator of academic achievement.

*Heavy episodic drinking.* Respondents were asked to indicate how many times during the preceding year they had “drunk so much that you clearly felt drunk”. Reponses were 0 = “0 times”, 1 = “1 time”, 3.5 = ”2–5 times”, 8 = “6–10 times”, 25 = “10–50 times”, and 50 = “more than 50 times”.

*Conduct problems.* Conduct problems were measured using 13 questions about different kinds of problematic behaviour during the preceding year, and categorized as suggested by Pedersen, Mastekaasa and Wichstrøm (2001). The first category was “serious conduct problems” and comprised the following question items: “stolen things worth more than NOK 1000”,^1^ “done vandalism or damage worth more than NOK 1000”, “purposely destroyed or broken such things as windows, bus seats, telephone booths, or mailboxes”, “broken in somewhere to steal something”, and “been in a fight using weapons”. The second category “aggressive conduct problems” comprised the items “had a violent quarrel with a teacher”, “sworn at a teacher”, “been called up to the headmaster for something wrong you had done”, and “been sent out of the classroom”. The last category “covert conduct problems” comprised the items “avoided paying for such things as movie theatres, bus rides or train rides or similar”, “skipped school one whole day”, “stolen things worth less than 500 NOK from a shop”, and “been away a whole night without informing your parents, or told your parents you were somewhere else than you really were”. Responses were made on a scale ranging from 0 to 50 times, however, they were dichotomized (yes = 1, no = 0) and the sum of scores for each category was used in the analysis.

### Statistics

Age, gender and grades predicted attrition between t1 and t2. In the analysis, this was corrected for by applying inverse probability weights. The distributions of scores on all measures apart from academic achievement were heavily skewed to the right. To avoid violation of the assumption of homoscedasticity in linear regression analysis, the natural logarithms of the scores were used in the analysis. Since there is no natural logarithm of zero, 0.1 was added to all values before conversion. The regression analysis was conducted using data from t1 and t2, and FD models, which may be explained as follows: The formula for OLS regression of a dependent variable on an independent variable at one time point is:

*DV*_*i*1_ = *β*_1_ * *IV*_*i*1_ + *β*_2_ * *C_i_* + *e*_*i*1_,

where DV is the dependent variable and IV is the independent variable for individual *i* at time 1. *C_i_* denotes other possible causes for DV that are time-invariant (i.e. they do not change over time). Similarly, the formula for OLS regression of a dependent variable on an independent variable at a second time point is:

*DV*_*i*2_ = *β*_1_ * *IV*_*i*2_ + *β*_2_ * *C_i_* + *e*_*i*2_.

Using OLS, the regression coefficient b_1_ will be biased if *IV*_*i*1_ and *C_i_* are correlated. However, with FD, the second formula is subtracted from the first, which eliminates *C*_i_. This yields an estimate of *β* that is not biased by time-invariant individual factors, since these are cancelled out from the regression analysis. In practice, FD simply involves regressing change from t1 to t2 in the dependent variable on change from t1 to t2 in the independent variable.

Using FD in the current study, depression, academic achievement, heavy episodic drinking and conduct problems were regressed on time spent gaming. In addition, depression, academic achievement, heavy episodic drinking and conduct problems were regressed on video game addiction.

### Ethics

The study procedures were carried out in accordance with the Declaration of Helsinki. The survey procedures were approved by the Norwegian Social Science Data Services. All students were informed of the purpose of the survey, and consented after having received this information. In addition, written consent was given by the parents of all students attending elementary school and junior high school.

## Results

Table 1 shows means, standard deviations and Spearman rank order correlations for the study variables. Time spent gaming at t1 was significantly and positively correlated with time spent gaming at t2. Video game addiction at t1 was significantly and positively correlated with video game addiction at t2. Time spent gaming was significantly and positively correlated with video game addiction (at both t1 and t2). Time spent gaming at t1 was significantly and negatively correlated with depression, academic achievement, serious CP, and aggressive CP at both t1 and t2, and significantly and negatively correlated with heavy episodic drinking at t1 (but not at t2) and covert CP at t2 (but not at t1). Video game addiction at t1 was significantly and positively correlated with depression at t1 but not at t2, significantly and negatively correlated with academic achievement at t1 and t2, and with heavy episodic drinking at t1 but not at t2. Video game addiction at t1 was significantly and positively correlated with serious, aggressive and covert CP at t1 and t2. Despite the fact that some of these correlation coefficients were statistically significant, it should be noted that they ranged from weak to moderate in effect size.

The results from the FD models are presented in Table 2. As all regression models except those including academic achievement included independent and dependent variables that were both logged, the coefficients are elasticities, which means that a 1% increase in the independent variable is associated with a percentage change equal to the coefficient in the dependent variable. Table 2 shows no significant associations between gaming amount and any of the dependent variables, except with aggressive CP, where the effect was of negligible magnitude. The effect sizes for video game addiction were, however, much larger and statistically significant. According to the models, a 10% increase in video game addiction is associated with 2.5% increase in depression, a 1.7 point decrease in average grades, 3.3% increase in serious conduct problems, 5.9% increase in aggressive conduct problems and 5.8% increase in covert conduct problems. Video game addiction was not, however, associated with frequency of heavy episodic drinking.

**Table 1.** Means, standard deviations *(SD)* and Spearman rank order correlation coefficients for all the study variables

Variable	Mean	*SD*	1	2	3	4	5	6	7	8	9	10	11	12	13	14	15
1. Gaming amount t1	25.23	44.02	–														
2. Gaming amount t2	23.56	46.51	.61	–													
3. Video game addiction t1	1.47	0.62	.67	.50	–												
4. Video game addiction t2	1.37	0.61	.52	.68	.53	–											
5. Depression t1	1.81	0.66	–.07	–.07	.12	.*00*	–										
6. Depression t2	1.89	0.73	–.08	–.05	.*03*	.11	.42	–									
7. Academic achievement t1	4.00	0.75	–.12	–.10	–.09	–.08	–.06	–.*03*	–								
8. Academic achievement t2	3.98	0.80	–.11	–.10	–.07	–.11	–.*04*	–.08	.64	–							
9. Heavy episodic drinking t1	2.02	5.38	–.06	–.*04*	–.05	–.06	.20	.08	–.12	–.04	–						
10. Heavy episodic drinking t2	2.42	4.72	–.*03*	–.*04*	–.*04*	–.05	.16	.13	–.05	–.*03*	.51	–					
11. Serious CP t1	0.33	0.69	.17	.17	.18	.17	.16	.09	–.12	–.10	.22	.15	–				
12. Serious CP t2	0.13	0.51	.12	.13	.09	.13	.06	.14	–.07	–.09	.11	.19	.24	–			
13. Aggressive CP t1	0.86	1.15	.20	.15	.21	.17	.15	.06	–.18	–.16	.27	.19	.44	.19	–		
14. Aggressive CP t2	0.32	0.75	.12	.15	.10	.18	.05	.13	–.10	–.13	.07	.21	.24	.38	.32	–	
15. Covert CP t1	0.80	0.99	–.*00*	–.*00*	.07	–.*00*	.30	.15	–.18	–.14	.46	.32	.33	.15	.42	.14	–
16. Covert CP t2	0.66	0.93	.02	.06	.07	.10	.17	.26	–.07	–.14	.24	.37	.19	.29	.21	.35	.35

*Note:* Coefficients in italics are not statistically significant at the *P* < 0.05 level.

**Table 2.** Depression, academic achievement, heavy episodic drinking and conduct problems (CP) regressed on gaming amount and video game addiction using first difference regression models

	Depression	Academic achievement	Heavy episodic drinking	Serious CP	Aggressive CP	Covert CP
Gaming amount	0.00	–0.00	–0.02	0.01	0.05^*^	0.03
Video game addiction	0.25^**^	–0.17^**^	–0.05	0.33^*^	0.59^**^	0.58^**^

*Note:* All variables are logged except academic achievement. ^*^P < 0.01, ^**^P < 0.001. Weighted for gender, age and academic achievement.

## Discussion

The level of gaming addiction in the current study was comparable with previous studies using the same measurement instrument. The mean score in our sample (1.47 at time 1, and 1.37 at time 2) was similar to what was reported for two samples of adolescent gamers in the Netherlands (1.52 and 1.54) (Lemmens et al., 2009). It was also similar to what was reported for the adolescent population in Germany (mean = 1.46) (Festl, Scharkow & Quant, 2013).

The current study showed that video game addiction was associated with higher level of depression, poorer academic achievement, and more conduct problems. This is in line with several other studies that have investigated the possible detrimental effects over time of experiencing problems with video games (Gentile et al., 2011; Lemmens et al., 2009; Mentzoni et al., 2011; Ream et al., 2011; Rehbein et al., 2010; Skoric et al., 2009). However, the associations between time spent gaming and negative outcomes were negligible. These findings are not in line with some previous studies (Anand, 2007; Gentile et al., 2004; Holtz & Appel, 2011; Lemona et al., 2011; Ream et al., 2011), but our findings support research that favours the growing notion that strong engagement with video games is not necessarily associated negative outcomes, and that researchers need to differentiate between strong engagement with games and video game addiction (Brunborg et al., 2013; Charlton & Danforth, 2007; Desai et al., 2010; Ferguson et al., 2011, 2012; Rehbein et al., 2010; Skoric et al., 2009; von Salisch et al., 2011).

Despite the strengths of the current study, there are several limitations that need to be addressed. Although the sample size was large compared to other studies in this field, there was high attrition between t1 and t2, and the subsample that did participate at both time points may not be representative of the Norwegian adolescent population. Some correction was made for this issue by weighting the data for gender, age and academic achievement, but care should be taken in generalizing the findings beyond the study sample. Secondly, while the first differencing method avoids omitted variable bias resulting from time-invariant individual effects, it does not control for the possible effect of time *variant* omitted variables. Therefore, it is possible that the observed effects are indirect and biased by unknown intermediate variables. Thirdly, all the information used in the study was self-reported and is therefore vulnerable to self-report bias. Fourth, we did not differentiate between different kinds of video games. It may be the case that some games may be more closely related to negative outcomes, while the opposite is true for other types of games. Therefore future research may benefit from differentiating between different types of games. Fifth, because our study lacks experimental design, it is not possible to be conclusive about the directionality of causation. It could be the case that for instance depression causes problems with video games rather than the opposite. It can also be the case that the relationship is reciprocal with an elusive starting point, and constitutes a downward spiral where video game addiction causes depression which in turn causes video game addiction. This form of relationship may also be true for those between academic achievement, conduct problems and video game addiction.

Despite these limitations that should be addressed in future research, the implications of the current study are firstly that the research in this field may benefit from continuing to differentiate between time spent on games and video game addiction. Our results indicate relatively strong relationships between gaming addiction and negative outcomes, and that these relationships are not spurious. This has implications for future studies that aim at establishing causal links between video game addiction and negative outcomes. The current study also contributes to fulfilling the proposed requirements for establishing causation in epidemiological studies (Hill, 1965), but see Rothman, Greenland and Lash (2008) for a critical appraisal of these criteria. The relationships between dependent and independent variables are fairly strong, our findings are consistent with previous studies, the associations are present after controlling for confounding factors (specificity), there appears to be a linear relationship (biological gradient), and the results are theoretically plausible. However, there is lack of studies that can establish the temporal order of cause and effect. Future longitudinal studies would be beneficial for determining such temporality. There is also lack of experimental studies that should be conducted in order to investigate more closely the causal mechanisms. Finally, studies that investigate the coherence between findings from future experimental and epidemiological studies are required.

In summary, the results from current study showed that video game addiction is associated with depression, decreased academic achievement, and with conduct problems, but it is not associated with heavy episodic drinking. However, the study also showed that the amount of time spent gaming is only negligibly associated with the same outcomes. These findings have implications for future research that aims at establishing causal links between video game addiction and negative outcomes.

## Footnotes

^1^ 8 NOK equals about 1 EUR.
